# Consumers' salient beliefs regarding dairy products in the functional food era: a qualitative study using concepts from the theory of planned behaviour

**DOI:** 10.1186/1471-2458-11-843

**Published:** 2011-11-03

**Authors:** Deborah J Nolan-Clark, Elizabeth P Neale, Yasmine C Probst, Karen E Charlton, Linda C Tapsell

**Affiliations:** 1 Smart Foods Centre, University of Wollongong, Wollongong, New South Wales, Australia; 2 School of Health Sciences, University of Wollongong, Wollongong, New South Wales, Australia; 3 Illawarra Health and Medical Research Institute, University of Wollongong, Wollongong, New South Wales, Australia

## Abstract

**Background:**

Inadequate consumption of dairy products without appropriate dietary substitution may have deleterious health consequences. Social research reveals the factors that may impede compliance with dietary recommendations. This is particularly important given the recent introduction of functional dairy products. One of the challenges for public health professionals is to demonstrate the efficacy of nutrition education in improving attitudes toward nutrient rich foods. The aim of this study was to explore the salient beliefs of adult weight loss trial participants regarding both traditional and functional dairy products and to compare these with a control group not exposed to nutrition education.

**Methods:**

Six focus groups were conducted, three with weight loss trial completers (*n *= 15) that had received nutrition education and three with individuals from the same region (*n *= 14) to act as controls. Transcribed focus groups were coded using the Theory of Planned Behaviour theoretical framework.

**Results:**

Non-trial participants perceived dairy foods as weight inducing and were sceptical of functional dairy products. A lack of time/ability to decipher dairy food labels was also discussed by these individuals. In contrast trial participants discussed several health benefits related to dairy foods, practised label reading and were confident in their ability to incorporate dairy foods into their diet. Normative beliefs expressed were similar for both groups indicating that these were more static and less amenable to change through nutrition education than control and behavioural beliefs.

**Conclusions:**

Nutrition education provided as a result of weight loss trial participation influenced behavioural and control beliefs relating to dairy products. This study provides a proof of concept indication that nutrition education may improve attitudes towards dairy products and may thus be an important target for public health campaigns seeking to increase intake of this food group.

## Background

Dairy products represent an important core food group within many Western diets. Items such as milk, cheese and yoghurt provide an important source of protein and are the greatest contributor of calcium to the Australian diet [1]. Dairy food intake has also been associated with a decreased risk of developing osteoporosis [2], and the metabolic syndrome [3]. Current public health recommendations suggest that Australian adults consume a minimum of two to three serves of dairy products per day for optimal health [4]. However, up to four serves of dairy foods per day may be recommended for particular population groups to ensure optimal calcium intake of a minimum of 1000 mg/day according to Australian Nutrient Reference Values [5]. In a large longitudinal study of Australian women aged 50-55 years, only 32% were found to be consuming a minimum of two serves of yoghurt, milk or cheese per day [6]. The latest available National Nutrition Survey indicates that Australian adults on average are not meeting recommended dietary intakes for calcium [7], potentially increasing the risk of developing osteoporosis in these individuals [3].

Social research enables exposure of the factors that may impede compliance with dietary recommendations. Within this research paradigm, the technique of focus groups provides a means for generating hypotheses relating to consumer behaviour [8]. For example, research on dairy product consumption in elderly and young women in New Zealand found that a fear of weight gain, perceptions of adverse health effects and inappropriate medical advice were important barriers to increasing consumption [9]. Whether this is also the case in the current Australian context is not known. It is an area worthwhile pursuing, particularly with the introduction of functional dairy products. Whilst a universally agreed upon definition of functional foods is lacking, in general functional foods may include those that have been formulated to provide additional health benefits beyond the provision of nutrients [10]. Functional dairy foods may include calcium enriched or plant sterol and omega 3 fortified products. These products also represent a key area of growth for the national dairy industry.

In order to interpret consumer opinions in a sound theoretical framework, it is necessary to articulate the analytical reference points. Such positions are provided by the theory of planned behaviour (TPB) which is widely utilised to predict an individual's likelihood of adopting a particular behaviour. It is an extension of the theory of reasoned action, by taking into consideration an individual's perceived self efficacy or sense of control to allow the performance of a particular behaviour [11].

The TPB implies that an individual's intention to perform a behaviour is influenced by one's attitude towards adopting the behaviour, an evaluation of the subjective norms or social influence of others who may encourage/discourage such a behaviour and an individual's perception of the level of control in their ability to adopt the behaviour [11].

Shaping these attitudes are salient beliefs including behavioural beliefs (reflecting attitudes towards the behaviour), normative beliefs (reflecting the social influences of others relevant to the behaviour and an individual's motivation to comply with such an influence) and control beliefs (reflecting those beliefs which underlie the perceived level of ease or difficulty one might experience towards adopting the behaviour) [11]_. _The TPB has previously been utilised to predict behaviour related to food choice [12] and has been validated to predict consumption of dairy products [13].

One of the challenges for public health professionals is to demonstrate the efficacy of nutrition education in improving attitudes toward nutrient rich foods. In particular, there is a lack of evidence evaluating the role that nutrition education may have on shaping attitudes towards dairy foods. It is important to evaluate these attitudes in Australian adults, as this group have reported inadequate calcium [7] and dairy product intake [6]. In this study we explore the uptake of nutritional advice to participants in a weight loss trial where the dietary guidance was based on the Australian Guide to Healthy Eating [4] in which dairy foods were a key component. A comparative group of similar non trial participants from the same study population was recruited identify differences of opinion which could be attributed to trial participation and exposure to nutrition education. We hypothesized that trial participants would express more positive attitudes and beliefs towards traditional dairy foods and new functional dairy products in comparison to non trial participants.

The aim of the study was to explore the salient beliefs underlying attitudes towards traditional and functional dairy products.

## Methods

Focus group interviews were conducted to ascertain salient attitudes and beliefs of participants regarding dairy products in general, current dairy product consumption recommendations and functional dairy products.

### Participants

Participants consisted of, individuals who had received intensive nutrition education following participation in a weight loss trial inclusive of 6 dietary counselling sessions with qualified dietitians. Education relating to traditional dairy products was provided over a twelve month period [14]. In particular participants were advised to consume reduced fat dairy products or an appropriate dietary alternative in quantities consistent with the Australian Guide to Healthy Eating [4] as part of a whole of diet approach to facilitate weight loss. In addition, many of the study participants that were randomized to consume a dietary prescription rich in omega 3 polyunsaturated fatty acids as part of the weight loss trial, were provided with specific advice relating to functional (omega 3 enriched) dairy products. Further details on the clinical trial from which the participants were drawn from can be found at http://www.anzctr.org.au, (ACTRN12608000425392).

A convenience sample of university employees drawn from the same region and matched for sex was recruited to act as a comparative group representative of individuals that had not received formal nutrition information. All trial participants who had completed the trial as of November 2009 were invited to participate in the study via an email and follow up phone call. University staff members were recruited via an email that was sent to all general staff, with interested parties receiving follow up phone calls to arrange session times.

All participants were provided with an information form outlining the study procedure and food of interest to be discussed and parking vouchers were provided. The study was approved by the University of Wollongong Human Research Ethics Committee and signed informed consent was provided by all participants prior to the commencement of the study. Data on demographic and anthropometric characteristics of participants including age range, highest level of education and estimated height and weight were collected on entry to the study.

### Focus group procedure

**Figure 1:**
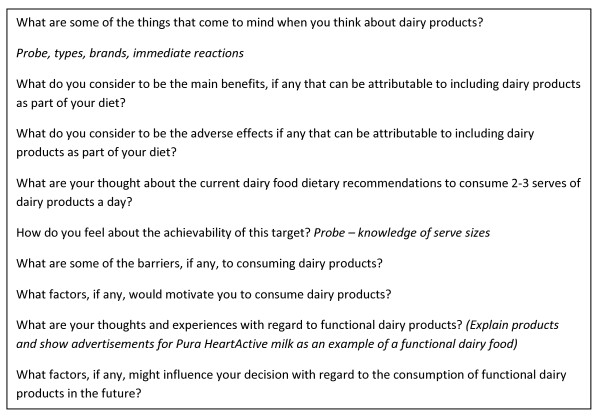
**Focus group questions to determine consumer perceptions of traditional and functional dairy products**.

Six semi-structured focus group interviews were conducted at the University of Wollongong in November 2009, within two months of participants completing the weight loss trial. All questions relating to dairy products were conducted by a trained moderator in the presence of an observer. The same moderator and observer facilitated each of the interviews to reduce any potential ambiguity in the results obtained. Interview questions were developed in accordance with general guidelines provided by Krueger and Casey [15] and influenced by themes identified throughout the literature. Interview questions were reviewed by members of the research team (DN, EN and LT) to ensure appropriateness to the topic of interest. Participants were encouraged to openly discuss their responses until no further views were expressed. Probing questions were utilised where required to encourage participants to clarify or expand on views expressed (Figure 1). University staff and trial participants attended separate focus groups to ensure that beliefs expressed could be attributed to the study group.

### Data analysis

All focus groups were recorded using two digital tape recorders and transcribed verbatim by the moderator (DN), with codes replacing participant names to ensure anonymity. Transcribed data were coded using the qualitative software package NVivo 7.0 (QSR International Pty Ltd, Melbourne, Australia, 2007). Coding was based on a method previously utilised by Patch et al [16] to describe consumer's salient beliefs on omega-3 enriched functional food products. This meant data were coded according to the TPB model into three distinct categories that constitute an individual's behavioural intention with regard to dairy product consumption. Categories of analysis consisted of *behavioural beliefs*, those beliefs which pertained to influence an individual's attitude towards dairy products, *normative beliefs*, an individual's consideration of the social pressure to consume/not consume dairy products based on the influences of peers and *control beliefs*, an individual's perceived efficacy in their ability to consume dairy products. Identified themes were discussed with several members of the research team (DN, EN and LT) until a consensus was reached regarding key thematic findings. All data outcomes were checked by the focus group observer (EN) to ensure accuracy.

An independent t-test was utilised to determine whether any between group differences existed for body mass index (BMI). All statistical analysis was performed using SPSS for Windows (version 17.0, SPSS Chicago, IL, 2008).

## Results and discussion

A total of 15 trial participants attended one of three focus groups. Three trial participants failed to attend their allocated focus group, citing time constraints as the reason for their absence. Fourteen non trial participants were recruited and all participated in one of the focus groups, resulting in a sample size of 29. Saturation of key themes was achieved throughout both study groups. A total of six focus groups of approximately 60-80 min duration were conducted. The study sample was predominantly female in both the trial participants and the non trial participants (Table 1). The trial participants were overweight on average with a mean BMI of 29.0 ± 4.0 kg/m^2^. This was significantly higher than the non trial participants with a mean BMI of 24.9 ± 5.0 kg/m^2 ^(*p *= 0.023).

**Table 1:** Focus group participant characteristics

	Trial Participants (*n *= 15)	Non-trial Participants (*n *= 14)
Gender frequency:		

-females	12	11

-males	3	3

Age frequency:		

- < 40 years	0	4

> 40 years	15	10

Mean BMI (kg/m^2^)	29.0	24.9

Education level Frequency:		

-Year 10-12	4	2

-Technical and Further Education (TAFE)	6	4

-University degree	5	8

### Salient behavioural beliefs

Overall non-trial participants reported that they believed that calcium and bone health were the key health benefits attributable to the consumption of dairy foods, with few other health benefits identified.

*"Your calcium comes to mind, yeah, it's drummed into you, your growing bones and I think for women like later on in life osteoporosis and those kinds of things..." *(Group 1, F, non trial participant)

This perception mirrors previous focus group outcomes reported by Hagy et al [17], whereby women attributed the health benefits of dairy foods to their calcium content and potential for osteoporosis prevention, and failed to identify other nutrients such as protein or specific vitamins and minerals present within dairy products. In contrast, the trial participants identified health benefits attributable to dairy products beyond bone health. Dairy foods were seen as a significant source of protein, vitamins and minerals. This may reflect the extent of nutrition education received by this group.

Beliefs relating to adverse health effects of dairy products were identified by several members of the non trial group who reported that they felt that these foods were fattening and mucous producing. The perception of dairy products as fattening throughout the control group is consistent with focus group findings by Hagy et al [17] and Eddy et al [18] and represents a challenge for nutrition professionals, particularly with current public health recommendations to consume a reduced fat diet [4]. This finding does not reflect literature in the nutrition science domain which suggests a potential weight loss effect associated with the consumption of dairy products [19]. Furthermore dairy fat has been associated with favourable metabolic profiles in large population studies [20-22].

Overall trial participants did not report perceiving dairy products to be weight inducing, with one participant discussing literature which indicated that dairy products may assist with weight loss. Most trial participants did not feel that the fat from full cream dairy products was cause for concern, discussing negligible differences between the fat content of full cream dairy products and their reduced fat counterparts. Another participant expressed the belief that reduced fat dairy products were high in sodium; however this view was not shared by the group. Persistent belief of fallacies surrounding dairy products, despite intensive nutrition education illustrates the difficult and challenging nature of communicating nutrition concepts related to food.

The idea that dairy products have been recently positioned in a negative way was discussed by both groups of participants. However, unlike the non trial participants that felt this 'demonisation' was due to the high fat content of dairy products, trial participants felt that dairy products were an easy target for this negative image for many individuals trying to distract themselves from other unhealthy dietary behaviours.

*"..It's a scapegoat, because we eat too much, don't exercise enough and there's too many fast food options, something had to be modified in this incredible way to make us all feel better..." *(Group 4, F, trial participant)

Discussions relating to functional dairy products by non trial participants indicated that most were highly resistant to these products. A high degree of scepticism with regards to their efficacy in producing beneficial health effects and the removal of the product from its natural state were cited as key factors which would impede consumption of these items for this group. The perception that dairy products have deviated significantly from their natural state over the years was also shared by trial participants, with a preference for 'natural' products shared by the group. However, trial participants expressed a more open attitude to consuming functional dairy products such as calcium enriched milk and dairy products containing plant sterols than non trial participants. This observation may reflect the exposure to what would be termed functional products by many participants throughout the clinical trial. Despite being more open to functional products than non trial participants, some degree of scepticism of their efficacy was also expressed by trial participants and a preference for food sources from which to derive additional health benefits naturally was discussed. Preferences for food products with minimal processing have been reported throughout the literature [23]. The perceived healthfulness of food products is an important factor influencing their acceptance by consumers and a food's health value is hypothesised to decrease in proportion with processing technologies it may have undergone [23]. Thus despite an overall improvement in attitude towards functional foods evident within the trial participants, preferences for natural food products with minimal processing was overriding.

### Salient control beliefs

Trial participants overall had considerably less perceived control barriers than non trial participants in relation to dairy product intake, with participants expressing an understanding of both dairy food serve sizing and recommended intakes of dairy products. Most trial participants practised label reading and could clearly articulate how they would incorporate the recommended amount of dairy foods into their diet. In contrast, non trial participants expressed a lack of knowledge about what constitutes a serve of dairy food and how many serves per day were recommended.

*"I have no idea what a serve really is" *(Group 3, M, non trial participant)

A lack of time and ability to decipher dairy food labels and health claims was also discussed by most group members that had not participated in the trial.

Access to dairy products was not perceived to be a barrier for their consumption by both groups as most participants felt they were freely available and readily accessible. However the relatively short shelf life of dairy foods was a concern for three non trial participants.

Despite these differences, several salient control beliefs remained consistent between both groups. Taste was an important factor influencing consumption of dairy foods, with preferences for full cream or reduced fat varieties expressed throughout both groups. Health issues including lactose intolerance, high cholesterol and lactose intolerance were discussed in relation to restricting dairy food intake by group members.

The high cost to perceived benefit of functional dairy products was a key belief expressed by non trial participants that may impede consumption of these items. A lack of trust in the efficacy of products available was discussed with reference to functional dairy items. Non trial participants were not convinced that these items actually contained the additional ingredients advertised or that addition of such ingredients would have any influence on their health status. The high cost of functional dairy products remained another consistent topic of discussion for trial participants, particularly given participants' lack of knowledge about the dosage of functional ingredients required to elicit a health benefit.

*"I'm very sceptical cos in the end say like you pay two dollars for the normal milk, the added calcium will cost you another dollar and is it really worth it?" *(Group 5, M, trial participant)

In a study in Northern Ireland the cost of functional or health enhancing dairy products was also identified as an issue for consumers, with only 35% of questionnaire respondents reporting feeling satisfied with the price of such products [24].

Perceived behavioural control has been reported in the literature to be most predictive of intention to consume dairy products and correlated significantly (*r *= .48, *P *< .001) with dairy product consumption in a sample of older adults [13]. Similarly, Park & Ureda [25] reported that pregnant women's perceived level of control was related to eventual milk consumption. Thus improvements in perceived efficacy regarding one's ability to consume dairy products or functional dairy foods may be the most important aspect of the TPB in terms of generating the desired behaviour. Overall trial participants discussed considerably less control barriers that may restrict dairy food consumption in comparison to non trial participants, indicating that nutrition education may have improved the beliefs surrounding dairy foods that are most likely to facilitate an increase in their eventual consumption.

### Salient normative beliefs

Family purchasing habits and preferences for dairy products were an important factor for both groups in terms of influencing dairy product consumption. Lactose intolerance within the family initiated the replacement of dairy foods with soy products for one participant, whilst a family history of osteoporosis was a key motivating factor for others to consume dairy foods. Several individuals discussed adding dairy products such as cheese and milk to evening meals in an effort to ensure that children were meeting calcium recommendations.

Government initiated school milk programs were also discussed as a normative influence that had either positively or negatively influenced milk consumption throughout both groups depending on the freshness of the milk provided. Trial participants in particular discussed feeling coerced to drink milk past its shelf life during the program.

*"They used to make you drink it hot and I haven't liked milk since" *(Group 5, F, trial participant)

This school milk program was introduced in 1951 as the Commonwealth's milk for school children Act and provided children under 13 with daily milk during school hours [26]. The scheme was introduced to improve the nutritional adequacy of children's diets, however it appears this scheme may have had some deleterious consequences in terms of milk acceptance in later years.

Several participants reported that health professionals such as general practitioners had discouraged consumption of items such as cheese due to its perceived high fat content and the replacement of full cream dairy products with their reduced fat counterpart.

*"..that was the first thing my doctor said when she looked at my body mass index, she said oh you must eat a lot of cheese, so that's from a GP" *(Group 2, F, non trial participant)

The normative influence of general practitioners in terms of identifying particular dairy products, such as cheese, as weight inducing or cholesterol raising was identified by both groups. Older women reported that physicians were the strongest normative influence regarding dairy foods with many avoiding particular items solely as a result of advice received from their doctor [18]. General practitioners are a key source of nutrition information for many individuals [27], thus any advice given should reflect the current body of evidence to limit unnecessary avoidance of key food groups. As expected, trial participants discussed that dietitians encouraged dairy product consumption or appropriate alternatives for individuals not willing to consume dairy foods.

In relation to functional dairy products, both groups reported feeling highly sceptical of the food industry and unlikely to purchase functional dairy foods based on advertising messages or their own initiative. Generally however, non trial participants would try these items if recommended by health professionals, whilst trial participants were sceptical of health practitioners recommending functional foods and wanted independent scientific research regarding the efficacy of these products to make their own judgements. Previous consumer research regarding perceptions of functional dairy products indicates that physicians and health professionals were regarded as being the most credible source of information regarding functional dairy foods [23]. Trial participants desire to seek independent scientific research may reflect their exposure to evidence based nutrition education that has fostered confidence in their ability to decipher nutrition messages autonomously. Alternatively, this contrast may reflect personality differences between the groups. The willingness of the trial group to volunteer for this study following completion of an intensive twelve month clinical trial suggests that these individuals were highly motivated with a desire to learn healthier eating practices.

Overall salient normative beliefs did not differ greatly between trial and control participants, potentially indicating that these influences are more static than attitudes and perceived behavioural control in relation to dairy foods. This is an interesting observation given that a study by Kim et al [13] found that behavioural and control beliefs in relation to dairy foods contributed to a predictive model of intention to consume dairy products, whilst normative beliefs did not influence this behavioural intention. Thus nutrition education appears to have been most efficacious in improving the belief types which have been demonstrated throughout the literature to result in increased dairy food intake.

### Limitations

Findings from this exploratory study should be interpreted with care due to the relatively small sample size of participants interviewed and the motivated nature of the trial participants. Thus, results may not reflect views of the general population. The two groups were not matched for weight status, with trial participants being overweight on average in comparison to non trial participants. This discrepancy may have influenced the outcomes observed. In addition it is likely that trial participants agreeing to take part in this study were those that had been successful with implementing the dietary changes suggested by trial dietitians. Thus the impact of nutrition education in this context for individuals that may not have completed the trial or did not achieve desired results was not able to be examined. However, the trial participants who took part in the present study were found to match closely with the broader clinical trial population, in terms of BMI and age and could thus be considered to be a representative sample of the population of interest.

Despite these limitations the important role of nutrition education in shaping consumers salient beliefs with regard to dairy product consumption is clearly visible. This is particularly the case with regard to the favourable influences on control and behavioural beliefs which have been reported to most influentially predict dairy product consumption [13].

Future research should quantify the impact of nutrition education with regard to influencing intent to consume both dairy products and functional dairy products using the TPB theoretical framework. Quantification of the role of nutrition education by appropriately qualified health professionals in predicting dairy product consumption may highlight the need to involve such individuals in the development and implementation of public health campaigns aimed at increasing dairy product consumption.

## Conclusions

Overall, key differences in salient control and behavioural beliefs in relation to dairy products were evident between weight loss trial participants and a comparative group that had not received nutrition education. Individuals that had received this education appeared more confident in their ability to incorporate dairy foods into their diet and to utilise label reading to identify appropriate products. A more favourable attitude towards dairy products in terms of their contribution to the diet and health effects was also observed in comparison to non trial participants.

Normative beliefs regarding dairy products were similar between the groups, indicating that these may be less amenable to change through nutrition education, and should possibly be a lower public health education priority when seeking to elicit change in dairy food consumption behaviour. Despite more favourable attitudes towards functional dairy products expressed by trial participants in comparison to non-trial participants, the degree of scepticism regarding these products retained by both groups suggests that nutrition education was insufficient to overcome control barriers such as the high cost for perceived benefit of these items in this sample. Whilst limited by its sample size, restricting generalisation, this exploratory study has provided a proof of concept demonstration that nutrition education that focuses on improving attitudes and self efficacy regarding the consumption of dairy products, will favourably influence salient beliefs that may translate to an improvement in eventual consumption of this core food group.

## Authors' contributions

DJN-C designed the study, moderated the focus groups, analysed the data and drafted the manuscript. EPN observed the focus groups, reviewed interview questions, discussed themes, checked data and contributed to the manuscript. LCT reviewed interview questions, discussed themes and contributed to the manuscript. YCP and KEC contributed to the manuscript. All authors read and approved the final manuscript.

## Competing interests

The authors declare that they have no competing interests.

## Pre-publication history

The pre-publication history for this paper can be accessed here:

http://www.biomedcentral.com/1471-2458/11/843/prepub
